# Some Observations on the Calcium Aluminate Carbonate Hydrates

**DOI:** 10.6028/jres.064A.032

**Published:** 1960-08-01

**Authors:** Elmer T. Carlson, Horace A. Berman

## Abstract

Two calcium aluminate carbonate hydrates, previously reported in the literature, were prepared and various properties determined. The compound 3CaO·Al_2_O_3_·CaCO_3_·11H_2_O, was prepared by precipitation from mixtures of solutions of calcium aluminate, calcium hydroxide and sodium carbonate; and, in less pure form, by several other means. It forms thin hexagonal plates having refractive indices 1.532 and 1.554. From X-ray diffraction, cell parameters, calculated on the assumption of hexagonal symmetry, are *a*=8.716, *c*=7.565. On continued exposure to atmospheric carbon dioxide it is decomposed to calcium carbonate and hydrated alumina.

The compound 3CaO·Al_2_O_3_·3CaCO_3_·32H_2_O, was prepared by precipitation from mixtures of ammonium bicarbonate, calcium aluminate, and calcium hydroxide in aqueous sucrose solution. It crystallized as spherulites of minute needles. Other methods produced individual crystals, some tubular, but only in minute quantities or mixed with other phases. X-ray diffraction indicates isomorphism with 3CaO·Al_2_O_3_·3CaSO_4_·32H_2_O. 3CaO·Al_2_O_3_·3CaCO_3_·32H_2_O is, in general, less stable than the monocarbonate complex.

The needle-form “silicoaluminate” reported by Flint and Wells was re-examined. It was concluded that the phase probably contains CO_2_ as well as SiO_2_, but the exact composition is in doubt.

## 1. Introduction

The existence of two series of calcium aluminate complex salts has been known for many years. One series may be represented by the general formula 3CaO·Al_2_O_3_·Ca*X*·*n*H_2_O, the other by 3CaO·Al_2_O_3_·3Ca*X*·*m*H_2_O, in which *X* is one of a number of divalent anions (or two units of a monovalent anion), *n* is 10 to 12, and *m* is about 30. The former series crystallizes typically as thin hexagonal plates, though the symmetry may be lower than hexagonal; the latter series usually occurs as long, slender needles. Perhaps the most familiar example of the first series is the calcium aluminate hydrate, 3CaO·Al_2_O_3_·Ca(OH)_2_·12H_2_O (more commonly written 4CaO·Al_2_O_3_·13H_2_O); whereas the acicular series is typified by the well-known aluminate sulfate, 3CaO·Al_2_O_3_·3CaSO_4_·32H_2_O.

The literature relative to this interesting class of compounds up to 1951, has been reviewed by Steinour [1]^1^. Some years earlier, at the Symposium on the Chemistry of Cement in Stockholm, F. E. Jones [2] summarized the existing data on the calcium aluminate complex salts, including two “carboaluminates” which had been prepared by G. E. Bessey. The formulas assigned were 3CaO·Al_2_O_3_·CaCO_3_·11H_2_O and 3CaO·Al_2_O_3_·3CaCO_3_·27H_2_O. More recently the low-carbonate compound was the subject of a study by Turriziani and Schippa [3] (who indicated the water content as 10.8 H_2_O), and its occurrence has also been reported by one of the present authors [4]. The high-carbonate compound has been prepared by Buttler [5], who found by X-ray methods that it is isomorphous with the “sulfoahiminate” of analogous composition.

It may be appropriate at this point to consider briefly the nomenclature of these compounds. The term “sulfoaluminate” has become firmly established through many years of usage, and it was therefore natural to refer to the more recently discovered carbonate analogs as carboaluminates. These names, unfortunately, do not correctly represent the relationship of the varous atoms involved. Although our knowledge of the crystal structure is incomplete, it seems clear that the sulfoaluminates might more correctly be called “aluminate sulfates”, and the analogous “carbo-aluminates”, “aluminate carbonates.” Thus, for example, the compound 3CaO·Al_2_O_3_·3CaCO_3_·nH_2_O (or 6CaO·Al_2_O_3_·3CO_2_·nH_2_O) would be called hexacalcium aluminate tricarbonate hydrate. Inasmuch as this terminology is rather cumbersome, the compounds will generally be referred to in this paper by formula rather than by name. The older terms sulfoaluminate and carboaluminate will be avoided except when needed in referring to earlier work.

The two aluminate carbonates are of some practical interest because of their possible relation to the chemistry of cement hydration. The compound 3CaO·Al_2_O_3_·CaCO_3_·11H_2_O has, in fact, been noted among the hydration products of aluminous cement [4, 6].

## 2. Procedures

The various reaction mixtures were prepared from reagent grade chemicals, except as noted below. Calcium hydroxide solutions were made from calcium oxide, freshly obtained by heating calcium carbonate. Calcium aluminate solutions were prepared by shaking monocalcium aluminate, CaO·Al_2_O_3_, with water for 30 to 40 min., followed by filtration. The anhydrous aluminate used for this purpose was made by heating a mixture of calcium carbonate and alumina (either hydrated or anhydrous) for several hours at about 1,250° C, and was used within a short time after cooling. In some cases, an aluminous cement was substituted for the CaO·Al_2_O_3_, and the shaking with water was continued for periods up to 3 hrs. By either method, concentrations up to 2.0 g Al_2_O_3_ and 1.2 g CaO per liter, or occasionally higher, were obtained. Such solutions are metastable, and usually start to become cloudy within the time required for an accurate analysis. An estimate of concentration based on titration with hydrochloric acid, in the presence of phenolphthalein, was therefore used as a basis for calculating the desired proportions in the mixtures, to permit use of the solution while still fresh. The actual concentration was later determined gravimetrically.

X-ray diffraction patterns by the powder method were made on a Geiger counter diffractometer, with copper K*α* radiation.

Infrared spectra were obtained on pressed pellets, with the sample in the amount of 0.3 to 0.4 percent dispersed in KBr. A Beckmann IK-4 infrared spectrometer with sodium chloride optics was used. The spectra include a small amount of absorption due to adsorbed water.

## 3. Results and Discussion

### 3.1. 3CaO·Al_2_O_3_·CaCO_3_·11H_2_O

The compound 3CaO·Al_2_O_3_·CaCO_3_·11H_2_O was prepared by mixing solutions of calcium aluminate, calcium hydroxide, and sodium carbonate, (or bicarbonate) following the method of Turriziani and Schippa [3]. The calcium aluminate solution was prepared from aluminous cement, as described in the previous section. Two liters of the aluminate solution and 8 to 9 liters of saturated calcium hydroxide were mixed and stirred a few seconds, after which sodium carbonate (3.50 to 3.75 g Na_2_CO_3_ in 250 ml of water) was added slowly with continued stirring. The mixture was allowed to stand about two months, after which the precipitate was filtered off and dried over saturated magnesium chloride solution (33% R.H.) until the water content, determined by ignition loss, was constant (requiring about 2 months). Details relative to two such preparations are given in table 1 (preparations 1.1 and 1.2). The molar ratios of these preparations, computed from results of analyses, are close to the theoretical composition 3CaO·Al_2_O_3_·CaCO_3_·11H_2_O. The small deviations probably are not quantitatively significant, as both preparations were observed to contain minute amounts of calcite (CaCO_3_), gibbsite (Al_2_O_3_·3H_2_O), and tricalcium aluminate hexahydrate (3CaO·Al_2_O_3_·6H_2_O).

The compound crystallizes in the form of minute hexagonal plates, which when viewed on edge appear to be needles showing positive elongation. In the preparations described above, the crystals were too thin to be distinguished except when observed edgewise. The low- and high-refractive indices were 1.532±0.003 and 1.554±0.003, respectively.

The X-ray diffraction pattern obtained for 3CaO·Al_2_O_3_·CaCO_3_·11H_2_O (preparation 1.1) is given in table 2. Certain weak reflections believed to be due to the known extraneous phases have been omitted from the tabulation. Assuming a hexagonal lattice, probable *hkl* values have been tabulated and the following unit cell parameters calculated: *a*=8.71_6_ A, ***c***=7.56_5_ A. The expected *d*-spacings are calculated from these parameters, and compared with both the observed *d*-spacings and the *d*-spacings published by Turriziani and Schippa [3]. It should be emphasized that the planes and parameters calculated in this manner are only a first approximation to the actual structure. Although there is good agreement between most of the observed and calculated *d*-spacings, those calculated for the *hkl* values referred to in footnote (c) of table 2 do not agree with the observed spacings as closely as the others. The true unit cell is probably larger and more complex, perhaps in the manner suggested by Buttler, Dent Glasser, and Taylor for 3CaO·Al_2_O_3_·Ca(OH)_2_·12H_2_O [7].

**Figure 1:**
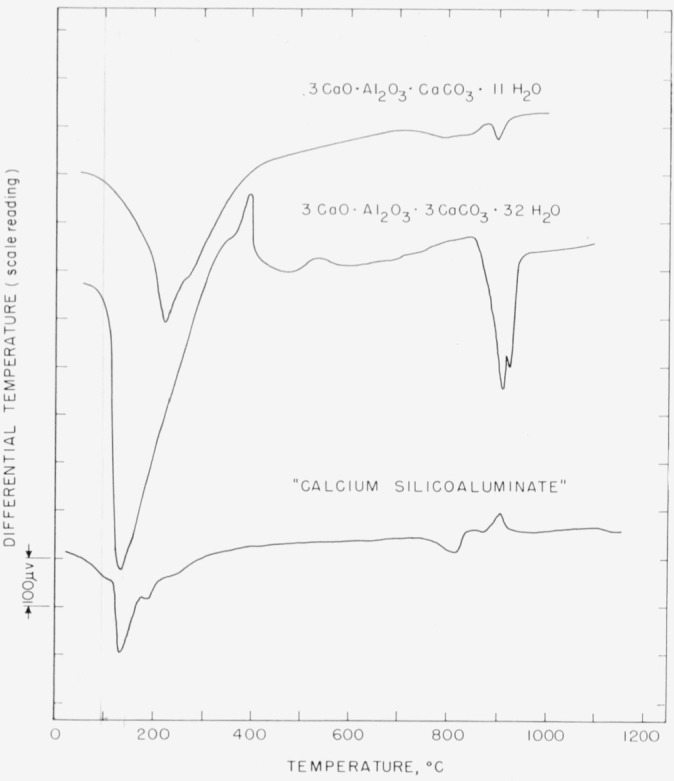
Differential thermal analysis curves for the calcium aluminate carbonate hydrates and silicoaluminate.

A differential thermal analysis curve for preparation 1.1 is shown in figure 1, top curve. The most notable feature is the endothermic effect at about 220° C, followed by a smaller one at 260° C. These are believed to be associated with the loss of water of hydration. The thermal effects at the higher temperatures are not as well defined, but it may be assumed that the carbon dioxide is expelled in this range. The departures here may be interpreted either as two endotherms separated by a brief recovery, or as a single broad endotherm culminating at 895° C, with a superimposed exotherm at 875°. The curve given by preparation 1.2 shows only minor deviations from that of 1.1, and both are similar to that given by Turriziani and Schippa [3].

**Figure 2:**
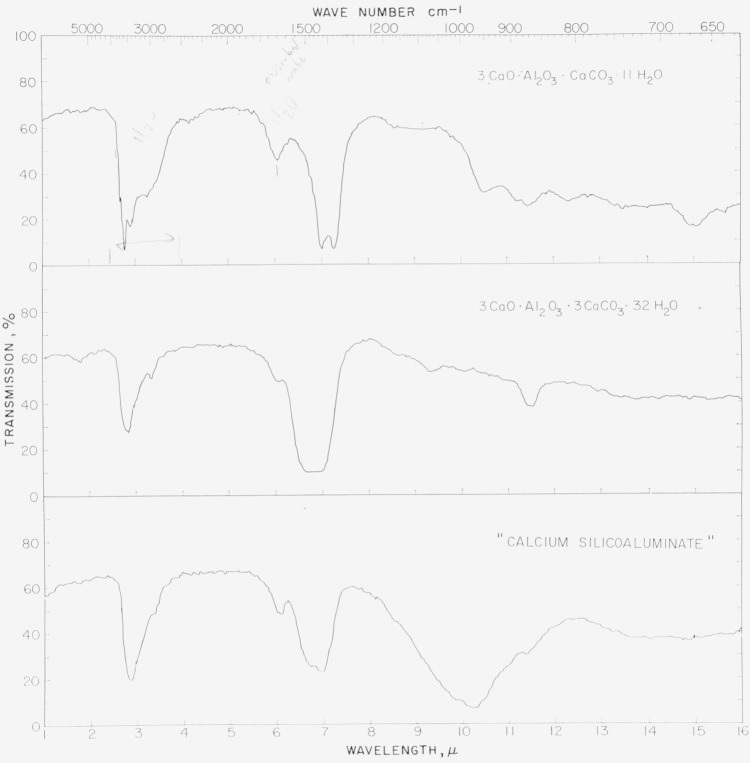
Infrared absorption spectra for the calcium aluminate carbonate hydrates and silicoaluminate.

A portion of the infrared absorption spectrum for preparation 1.1 is shown in the top curve of figure 2. While no attempt has been made to analyze the spectrum in detail, it may be noted that the multiple band between 2.5 and 4 *μ* and the smaller one at about 6.1 *μ* are probably due to combined or adsorbed water. The effect at 6.1 *μ* may be largely eliminated, and the one at lower wave length partially so, by preliminary heating of the sample at 110° C. It is therefore inferred that the effect at 6.1 *μ* is associated with adsorbed water, and the larger band at 2.5 to 4 *μ* with both combined and adsorbed water.

The double absorption band at 7.0 to 7.3 *μ* is believed to be associated with the carbonate group. Calcite and aragonite show a moderately broad band between 6.5 and 7.0 *μ*, from which it may be inferred that the bonding here is somewhat different. Heating the material at 110° C changes the doublet into a single band, but does not change its position. The rest of the spectrum is essentially unchanged by the heat treatment.

3CaO·Al_2_O_3_·CaCO_3_·11H_2_O was also prepared by exposing calcium aluminate solutions to atmospheric carbon dioxide, both at room temperature (25 to 30° C) and at 1° C. In no case was a pure preparation obtained in this manner, small amounts of calcium carbonate and hydrated alumina being formed at the same time. On continued exposure, the aluminate carbonate that had formed was entirely decomposed, the products being calcite, aragonite, gibbsite, and bayerite. The decomposition may be represented by the equation,

$$\begin{array}{c} 3\mathrm{CaO}\cdot {\mathrm{Al}}_{2}O_{3}\cdot {\mathrm{CaCO}}_{3}\cdot 11H_{2}O+3{\mathrm{CO}}_{2}\\ \to 4{\mathrm{CaCO}}_{3}+{\mathrm{Al}}_{2}O_{3}\cdot 3H_{2}O+8H_{2}O\\ \begin{array}{cc} (\mathrm{calcite}\;\text{or} & (\mathrm{gibbsite}\;\text{or}\\ \mathrm{aragonite}) & \mathrm{bayerite}) \end{array} \end{array}$$

The conditions favoring one form of calcium carbonate or alumina over the other were not investigated. The reactions were slower at 1° C than at room temperature, but apparently followed the same course.

From the foregoing, it is clear that 3CaO·Al_2_O_3_·CaCO_3_·11H_2_O in aqueous suspension is not stable in the presence of carbon dioxide at pressures as high as the partial pressure of carbon dioxide in ordinary air. If the compound has a range of stable existence it must be in equilibrium with carbon dioxide at lower pressure. The probability that it does have a limited region of stability is suggested by the following experiments. A mixture of finely ground anhydrous tricalcium aluminate and calcium carbonate in equimolar ratio was made up to a paste with water in a vessel cooled with ice water. The paste was packed into a glass vial and examined after 7 days moist storage at room temperature. The X-ray diffraction pattern of the hardened paste indicated almost complete conversion to 3CaO·Al_2_O_3_·CaCO_3_·11H_2_O, with very small amounts of calcite and 3CaO·Al_2_O_3_·6H_2_O. Similar results were obtained if calcium carbonate was present in excess; or if the mixture was shaken with excess water in a stoppered flask instead of being made into a paste; or if the temperature of storage was lowered to 1° C. The overall reaction may be simply represented by the equation,

$$\begin{array}{l} 3\mathrm{CaO}\cdot {\mathrm{Al}}_{2}O_{3}+{\mathrm{CaCO}}_{3}+11H_{2}O\\ \to 3\mathrm{CaO}\cdot {\mathrm{Al}}_{2}O_{3}\cdot {\mathrm{CaCO}}_{3}\cdot 11H_{2}O \end{array}$$

No attempt was made to determine the actual mechanism.

In another series of experiments, various calcium aluminate hydrates were covered with calcium hydroxide solutions of appropriate concentration and exposed to the atmosphere in unstoppered bottles for 10 weeks. The aluminates used were CaO·Al_2_O_3_·10H_2_O, 2CaO·Al_2_O_3_·8H_2_O, 4CaO·Al_2_O_3_·13H_2_O, and 3CaO·Al_2_O_3_·6H_2_O. All were altered completely during this period except 3CaO·Al_2_O_3_·6H_2_O at 1° C; in the latter case a small amount of the original material remained. The final products were chiefly calcite and bayerite, but aragonite and gibbsite also were present in some cases. 3CaO·Al_2_O_3_·CaCO_3_·11H_2_O was observed during the earlier stages except when CaO·Al_2_O_3_·10H_2_O was the starting material. It persisted longer in those held at 1° C than in those at room temperature, and in two cases there was still some present after 10 weeks.

In general, then, it appears that the calcium aluminate hydrates in contact with water are attacked by atmospheric carbon dioxide with the formation of 3CaO·Al_2_O_3_·CaCO_3_·11H_2_O, which in turn is decomposed to calcium carbonate and alumina. However, when atmospheric carbon dioxide is excluded, calcite can react with calcium aluminate to form 3CaO·Al_2_O_3_·CaCO_3_·11H_2_O. Presumably there is a point of equilibrium at some carbon dioxide concentration lower than that of the atmosphere, but this has not been investigated.

### 3.2. 3CaO·Al_2_O_3_·3CaCO_3_·32H_2_O

Several attempts were made to prepare 3CaO·Al_2_O_3_·3CaCO_3_·32H_2_O by the same technique used successfuly in preparing the monocarbonate complex; that is, from solutions of calcium aluminate, calcium hydroxide, and sodium carbonate. Preparations 1.3 and 1.4, table 1, are representative of a series in which the initial ratios of CaO and CO_2_ to Al_2_O_3_ were progressively increased. All these preparations showed the X-ray diffraction pattern and differential thermal analysis peaks of 3CaO·Al_2_O_3_·CaCO_3_·11H_2_O and calcite. The intensities of the calcite peaks relative to those of the aluminate complex increased with the calcium carbonate content of the preparations. Optical examination showed crystals of 3CaO·Al_2_O_3_·CaCO_3_·11H_2_O and very fine particles of calcite. No 3CaO·Al_2_O_3_·3CaCO_3_·32H_2_O was observed. Details of composition are given in table 1.

The other method employed, in which atmospheric carbon dioxide was allowed to react with calcium aluminate solutions, proved more successful. After numerous experiments it was concluded that the conditions favoring the formation of the needle-form compound are rather critical. Favorable results were obtained by the action of atmospheric carbon dioxide on a calcium aluminate solution containing 0.5 to 0.7g Al_2_O_3_ and 0.4 to 0.6g CaO per liter. Such solutions were prepared by the action of water on CaO·Al_2_O_3_, followed by dilution of the filtrate with water and calcium hydroxide solution. The diluted solution was cooled to 10 to 20° C, and air was bubbled through at a moderate rate. In a short time, usually about 20 min, the needle crystals could be observed, and at that point, more calcium hydroxide could be added to increase the yield. Despite all precautions, the preparations always contained either calcite or 3CaO·Al_2_O_3_·CaCO_3_·11H_2_O in small amounts. Increased contamination resulted if the solutions were not freshly prepared, if the temperature exceeded 20° C, or if the volume aerated exceeded 200 ml. It was therefore difficult to prepare more than a few milligrams of reasonably pure material at a time. The crystals obtained were long, slender needles, similar in appearance to those of 3CaO·Al_2_O_3_·3CaSO_4_·32H_2_O. The high and low indices of crystals that had been dried over magnesium perchlorate were 1.480 and 1.456. These may be compared with the indices 1.464 and 1.458 for the sulfoaluminate, reported by Lerch, Ashton, and Bogue [8]. The ratio of CaO to Al_2_O_3_ was in approximate agreement with the formula, but the amount of precipitate available was too small for an accurate determination of the CO_2_ content.

**Figure 3:**
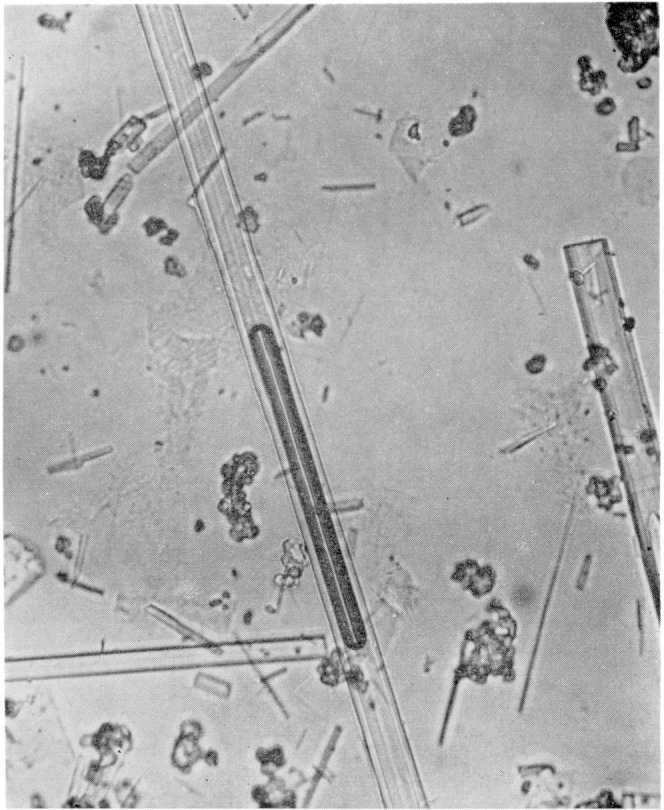
Photomicrograph showing tubular crystals of *3CaO·Al_2_O_3_·3CaCO_3_·32H_2_O* Magnification, × 310. Largest crystal contains entrapped bubble.

Small amounts of 3CaO·Al_2_O_3_·3CaCO_3_·32H_2_O were also formed when calcium aluminate solutions derived from an aluminous cement were allowed to stand overnight in open beakers. Such preparations always contained large amounts of 3CaO·Al_2_O_3_·CaCO_3_·11H_2_O as well as other crystals and amorphous material, but they were of interest because of the relatively large size of the needle crystals. In several instances the larger crystals were clearly tubular in shape, as may be seen in the photomicrograph, figure 3. In this picture, the gas bubble, which aids in discerning the tubular habit, presumably consists of ether vapor derived from the drying treatment. Such bubbles were gradually absorbed by the immersion liquid, and disappeared entirely in a few minutes.

For reasons not yet understood, the crystals formed in this manner had somewhat higher indices of refraction, namely, 1.490 and 1.470. The X-ray diffraction pattern, however, appeared to be identical with those of the previous preparation.

The difficulties attending preparation of 3CaO·Al_2_O_3_·3CaCO_3_·32H_2_O led to a modified procedure designed to permit a high concentration of CaO while inhibiting precipitation of CaCO_3_. The CaO was dissolved in aqueous solutions of sucrose (ordinary granulated sugar) ranging from 2 to 10 percent. After clarification by settling, the CaO concentration was determined by titration. A calculated volume of calcium aluminate solution was then poured into the lime-sucrose solution (no precipitate being formed at this point), after which a weighed quantity of ammonium bicarbonate, dissolved in water, was added. A flocculent precipitate formed, consisting of minute spherulites of uniform size, apparently formed of needles too small to be resolved by the microscope. Details of three such precipitation experiments are given in table 3.

The three products appeared identical under the microscope. Thus in this method the initial concentration of the solution appears not to be critical. It may also be noted that it was not necessary to cool the solutions below room temperature. From the analysis of preparation 3.3, after drying to constant weight over saturated ammonium chloride solution (79% R.H.), the composition was calculated to be 6.00 CaO:1 Al_2_O_3_:3.02 CO_2_:31.9 H_2_O, or in whole numbers, 3CaO·Al_2_O_3_·3CaCO_3_·32H_2_O. Approximately 3 molecules of water were lost on drying over magnesium perchlorate. The X-ray pattern of this preparation is given in table 4.

The differential thermal analysis curve (fig. 1, middle curve) shows, as its most prominent feature, a large endothermic effect at 135° C, evidently resuiting from expulsion of water of hydration. This endotherm is followed by an exotherm at 390°, the reason for which has not been investigated. The small exotherm near 500° likewise remains unexplained. The endotherm at 910° may be assumed to be caused by liberation of CO_2_, but the reason for the slight irregularity just above that point is not known.

The infrared absorption spectrum (fig. 2, middle tracing) shows some similarity to that of 3CaO·Al_2_O_3_·CaCO_3_·11H_2_O, but there are differences in detail. In both spectra there is a large absorption band with a maximum near 3*μ*, and a small one at 6.1*μ*. With both preparations, heating at 110° C diminished the effect at 3*μ* and eliminated that at 6.1*μ*. In both cases, the absorption bands may probably be ascribed to combined and adsorbed water.

In the spectrum for 3CaO·Al_2_O_3_·3CaCO_3_·32H_2_O, there is a large band at 6.5 to 7*μ*, also a smaller one near 11.5*μ*. These are similar in size and position to bands observed for calcite and aragonite, and attributed to the carbonate group. The relationship here appears to be closer than that between 3CaO·Al_2_O_3_·CaCO_3_·11H_2_O and calcite and aragonite.

### 3.3. Relation of 3CaO·Al_2_O·3CaCO_3_·32H_2_O to Other Compounds of the Same Type

#### a. 3CaO·Al_2_O_3_·3CaCO_4_·32H_2_O

The isomorphous relationship between 3CaO·Al_2_O_3_·3CaCO_3_·32H_2_O and the analogous sulfate compound has already been mentioned. The possibility of solid solution between the two seemed worth investigating, and a few experiments were directed toward that end. The problem was attacked from two angles. The first procedure consisted of partial substitution of carbonate for sulfate in mixtures known to yield 3CaO·Al_2_O_3_·3CaSO_4_·32H_2_O crystals. In every case, the product was a mixture of 3CaO·Al_2_O_3_·3CaSO_4_·32H_2_O and 3CaO·Al_2_O_3_·CaCO_3_· 11H_2_O, with no observable displacement in the X-ray pattern of either.

The other procedure involved substitution of ammonium sulfate for half of the ammonium bicarbonate in the lime-sucrose-solution method successfully used in preparing 3CaO·Al_2_O_3_·3CaCO_3_· 32H_2_O. A heavy precipitate was obtained but the crystals were so minute that their shape could not be ascertained. There appeared to be two phases, the predominant one having a mean index of refraction below 1.47, the other above 1.48. The X-ray diffraction pattern showed strong lines of 3CaO· Al_2_O_3_·3CaSO_4_·32H_2_O, together with very weak lines of 3CaO·Al_2_O_3_·3CaCO_3_ 32H_2_O. The results are not entirely conclusive, because of the extreme fineness of the precipitate and the very small differences between the respective indices of refraction and X-ray patterns. Nevertheless, the inference may be drawn that if any solid solution occurs, it is so limited in extent that it cannot be demonstrated by the methods used. The reason for the surprising difference in the relative amounts of the two phases has not been investigated.

#### b. Hexacalcium aluminate Hydrate

Preparation of a hexacalcium aluminate hydrate, 6CaO·Al_2_O_3_·33H_2_O (or 3CaO·Al_2_O_3_·3Ca (OH)_2_·30H_2_O) was reported by Flint and Wells [9] in 1944, but confirmation from other source appears to be lacking. The compound was prepared by mixing lime-sucrose solution with calcium aluminate solution; that is, the method was the same as described above for 3CaO·Al_2_O_3_·3CaCO_3_·32H_2_O except that no carbonate was added. In connection with the present study, an attempt was made to prepare the hexacalcium aluminateby following the method of Flint and Wells. A surprisingly small amount of precipitate was obtained, which had, however, a CaO:Al_2_O_3_ ratio close to 6, as expected. The optical properties approximated those reported by Flint and Wells, although the crystals were too small for a precise determination of indices of refraction. Because of the small amount of the precipitate, carbon dioxide was not quantitatively determined, but its presence was noted qualitatively. Further attempts at preparation of hexacalcium aluminate yielded still less precipitate, or none at all. The X-ray pattern of the precipitate proved to be identical with that of 3CaO·Al_2_O_3_·3CaCO_3_·32H_2_O from which it is inferred that the material actually was the tricarbonate complex. The carbon dioxide probably was derived from the lime-sucrose solution, which in that instance had been subjected to repeated filtration in an effort to remove the lime remaining in suspension, without exclusion of atmospheric carbon dioxide. Thus the existence of a hexacalcium aluminate hydrate was not confirmed.

#### c. Calcium Silicoaluminate

Flint and Wells [9] reported the preparation of two calcium silicoaluminates analogous to the sulfoaluminates. One of these, crystallizing as long, hairlike needles, appeared in a number of aqueous lime-alumina-silica mixtures that had been kept for three years or longer. It could not be separated from the other solid phases present for analysis, but because of its marked similarity to 3CaO·Al_2_O_3_·3CaSO_4_·32H_2_O it was tentatively assigned the formula 3CaO·Al_2_O_3_·3CaSiO_3_·30–32H_2_O. This phase formed slowly and progressively, while the mixtures were kept in rubber-stoppered glass flasks at room temperature over a period of years. Fortunately, four such mixtures, prepared by Flint and Wells, are still available for examination. One, containing a greater proportion of the acicular crystals than the others, was singled out for special study. In addition to the crystals, it contained a large amount of more or less gelatinous material with an average index of refraction about 1.51. Numerous attempts to separate the crystals from the gel were unsuccessful. The high and low indices of the needles were found to be 1.495 and 1.473, and the elongation was negative. These indices are slightly higher and lower, respectively, than those reported by Flint and Wells: *ω*=1.487; *ϵ* = 1.479. The discrepancy may result from different degrees of drying. Because of the radically different nature of the two types of material present, an accurate estimate of the relative amounts was impossible, but the needle phase appeared to be over 50 percent of the total. The X-ray diffraction record showed a strong pattern similar to that of 3CaO·Al_2_O_3_·3CaCO_3_·32H_2_O, (see table 3). In addition, there were weaker and more diffuse lines corresponding to 2CaO·Al_2_O_3_·SiO_2_·*n*H_2_O and to hydrated calcium silicate. These have been omitted from the table. The intensities of the latter reflections were smaller than might have been expected from the amount of gel-like material present, hence it must be concluded that this material is poorly crystallized.

By chemical analysis, the overall molar ratio of the nonvolatile components in the precipitate was found to be 5.48 CaO:1 Al_2_O_3_:2.81 SiO_2_. This could be construed to lend good support to the formula proposed by Flint and Wells, were it not for the other solid phases known to be present in significant quantity. Analysis also indicated 0.02 percent SO_3_, which was considered negligible. However, there was also a considerable amount of CO_2_, which presumably had permeated through or around the rubber stopper from the atmosphere during the 18-year period of storage. In terms of the molar ratio given above, it amounted to 0.73 mole per mole of Al_2_O_3_, which is too much to be ignored. Such a quantity of CO_2_, if combined as calcite or aragonite, would be conspicuous under the polarizing microscope and readily detectable by X-ray diffraction. Another possible form of combination, 3CaO·Al_2_O_3_·CaCO_3_·11H_2_O, likewise could hardly escape notice if present in the indicated proportion. Since none of these substances was observed, it would be reasonable to infer that the needle crystals contain the CO_2_ and that they actually are 3CaO·Al_2_O_3_·3CaCO_3_·32H_2_O. Unfortunately, this conclusion also fails to fit all the facts. The X-ray patterns show small, but significant, differences ; and the amount of CO_2_ found is inadequate to account for the large proportion of needle crystals present if the formula showing 3CaCO_3_ is accepted. The chemical analysis can be rationalized by the assumption of a quinary compound (possibly a solid solution) with a composition approximating 3CaO·Al_2_O_3_·1.5CaCO_3_·1.5CaSiO_3_·*n*H_2_O. It is recognized that this suggested formula rests on no firmer foundation than does that proposed by Flint and Wells, except that it does account for the CO_2_ shown to be present.

A differential thermal analysis curve for the preparation under discussion is shown in figure 1, bottom curve. In comparison with the curve for 3CaO·Al_2_O_3_·3CaCO_3_·32H_2_O, the endotherm corresponding to loss of water occurs at the same temperature, but that attributed to liberation of CO_2_ comes at about 815°, considerably lower than in the tricarbonate compound. This shift is probably due to the presence of silica. The exotherm at about 850° C may represent a lime-silica reaction, but the significance of the other irregularities is not known.

The infrared absorption spectrum (fig. 2, bottom) closely resembles that of 3CaO·Al_2_O_3_·3CaCO_3_·32H_2_O in the region of lower wave lengths. The wide absorption band extending from 8 to 12 *μ* is typical of hydrated calcium silicates.

The results described above are supported by less complete data for three other mixtures prepared by Flint and Wells. Two of them contained the same solid phases as the one discussed, but with the needle phase present in smaller amount. The other was predominantly 3CaO·Al_2_O_3_·CaCO_3_·11H_2_O, with lesser amounts of the needle phase and calcium silicate hydrate. The X-ray patterns showed the stronger lines of the needle phase, with no significant variation in *d*-spacings among the different preparations.

Similar acicular crystals have appeared in smaller amounts in at least 10 aqueous lime-alumina-silica mixtures prepared more recently by one of the authors. The crystals differed somewhat in habit, usually being shorter and thicker, but the indices of refraction were the same, within limits of measurement. It seems significant that in all cases, the acicular crystals were formed only after the mixtures had stood several months or years in rubber-stoppered flasks, thus affording opportunity for slow diffusion of CO_2_ from the atmosphere.

The conclusion, then, is that the acicular crystals in question are neither a pure aluminate silicate nor a pure aluminate carbonate, but may contain both CaCO_3_ and CaSiO_3_.

Quite recently, Mohri [10] reported the preparation of a compound which he believed to be the same as the silicoaluminate of Flint and Wells, 3CaO·Al_2_O_3_·3CaSiO_3_·31H_2_O. The product was obtained by hydrating lime-alumina-silica glasses at 20° C, with careful exclusion of CO_2_. However, the electron micrographs showed tabular rather than acicular crystals, and the X-ray diffraction patterns showed strong lines in agreement with the pattern of 2CaO·Al_2_O_3_·SiO_2_·*n*H_2_O. Consequently this work cannot be taken unreservedly as a confirmation of the conclusions of Flint and Wells.

### 3.4. Relation to the Chemistry of Cements

The possible relation of the aluminate carbonates to the hydration of hydraulic cements has scarcely been touched in the present study, but it seems appropriate to point out that the subject deserves further exploration. The components of these compounds are all present in the cements, the aggregates, the water, or the atmosphere to which concrete and mortar are exposed during and after mixing and placing. Hence the formation of aluminate carbonates in such materials, though perhaps only metastably, must be considered a possibility. The occurrence of 3CaO·Al_2_O_3_·CaCO_3_·11H_2_O in hydrated aluminous cement pastes has been noted above. Schippa [6] found this compound chiefly near the surface of the specimens, as might be expected. The formation of a similar crystalline phase at the interface between aluminous cement and calcareous aggregate was noted by Farran [11], who, however, considered it to be a solid solution between 3CaO· Al_2_O_3_·CaCO_3_·11H_2_O and 4CaO·Al_2_O_3_·13H_2_O. No report of the formation of such compounds in portland cement has been noted.

From the experimental work recorded above, it appears unlikely that 3CaO·Al_2_O_3_·3CaCO_3_·32H_2_O would be formed in hydraulic cements, except perhaps under rather unusual conditions. 3CaO·Al_2_O_3_·CaCO_3_·11H_2_O, however, should form readily, especially near the surface. Under continuous exposure to the atmosphere it would be expected to decompose with formation of calcium carbonate and alumina; but if formed beneath the surface, from reaction with calcareous aggregate, it might persist indefinitely.

## 4. Summary

Two calcium aluminate carbonate hydrates, previously reported in the literature, have been prepared and various properties determined.

3CaO·Al_2_O_3_·CaCO_3_·11H_2_O, crystallizes as thin hexagonal plates having refractive indices 1.532 and 1.554. The X-ray diffraction pattern has been tentatively indexed on the assumption of hexagonal symmetry, and the unit cell parameters calculated to be *a*=8.716, *c*=7.565. It was prepared by precipitation from mixtures of solutions of calcium aluminate, Ca(OH)_2_, and Na_2_CO_3_; by the action of atmospheric CO_2_ on calcium aluminate solutions, and on calcium aluminate hydrates in aqueous suspension; and by reaction between 3CaO·Al_2_O_3_,·CaCO_3_ and water, in paste form. On continued exposure to atmospheric CO_2_, it is decomposed to CaCO_3_ and hydrated Al_2_O_3_.

3CaO·Al_2_O_3_·3CaCO_3_·32H_2_O, crystallizes in the form of needles, which in some cases are shown to be tubular. The lower index of refraction ranges from 1.456 to 1.470, the higher from 1.480 to 1.490 in different preparations. It could not be prepared by the method used most successfully in obtaining 3CaO·Al_2_O_3_·CaCO_3_·11H_2_O; that is, by precipitation with Na_2_CO_3_. Precipitation by the action of atmospheric CO_2_ on calcium aluminate solutions was successful only over a closely restricted range of concentration, temperature, and volume. A more satisfactory procedure was the precipitation by ammonium bicarbonate from a sucrose solution containing calcium aluminate and relatively high concentrations of Ca(OH)_2_. 3CaO·Al_2_O_3_·3CaCO_3_·32H_2_O is, in general, less stable than 3CaO·Al_2_O_3_· CaCO_3_·11H_2_O.

Attempts to prepare solid solutions between the aluminate carbonates and the isomorphous aluminate sulfates were unsuccessful. The same is true regarding attempts at preparation of the hexacalcium aluminate hydrate described by Flint and Wells.

The silicoaluminate, 3CaO·Al_2_O_3_·3CaSiO_3_·30–32H_2_O, reported by Flint and Wells, has been reexamined. Although the composition still has not been established, it is concluded that the phase in question probably contains CO_2_ as well as SiO_2_.

## Figures and Tables

**Table 1:** Preparation of *3CaO·Al_2_O_3_·CaCO_3_·11H_2_O*

Preparation No.	1.1	1.2	1.3	1.4

Total volume of mixture liter Original concentration of mixture, calculated:	10.2	10.2	5.6	5.8
CaO g/liter	1.129	1.158	1.142	1.147
Al_2_O_3_ g/liter	0.358	0.355	0.347	0.240
Na_2_CO_3_ g/liter	0.342	0.337	0.519	0.705
Molar ratio, CaO: Al_2_O_3_	5.74	5.93	5.98	8.70
Molar ratio, CO_2_: Al_2_O_3_	0.92	0.91	1.44	3.06
Final concentration of solution:^a^				
CaO g/liter	0.387	0.414	0.291	0.328
Al_2_O_3_ g/liter	0.007	0.008	0.007	0.005
Molar ratios in precipitate (dried at 33% R.H.):				
CaO: Al_2_O_3_	4.05	3.96	4.43	5.80
CO_2_: Al_2_O_3_	1.02	0.98	1.57	3.47
H_2_O: Al_2_O_3_	10.6	10.7	11.0	9.1
Solid phases in precipitate	Nearly all 3CaO·Al_2_O_3_· CaCO_3_·11H_2_O. Very small amounts of calcite and 3CaO·Al_2_O_3_·6H_2_O.	Same as 1.1	3CaO·Al_2_O_3_·CaCO_3_·11H_2_O and calcite.	3CaO·Al_2_O_3_·CaCO_3_·11H_2_O and calcite

a Other constituents not determined.

**Table 2:** X-ray diffraction pattern of *3CaO·Al_2_O_3_·CaCO_3_·11H_2_O*

Observed *d*-spacings	Relative intensity	Probable *hkl*^a^	Calculated *d*-spacings^a^	*d*-spacings observed by T and S^b^	Remarks

7.567	100	$\{\;\begin{array}{c} 001\\ 100 \end{array}$	7.565 7.548	} 7.740	
4.360	6	110	4.359	…………	May be a line of Al_2_O_3_·3H_2_O.
3.985	6	…………	…………	…………	
3.784	44	$\{\;\begin{array}{c} 002\\ 111\\ 200 \end{array}$	3.783 3.776 3.774	} 3.790	
3.661	2	…………	…………	…………	
3.458	6	…………	…………	…………	
2.858	30	$\{\;\begin{array}{c} 112\\ 210 \end{array}$	2.857 2.853	} 2.890	
2.777	8	…………	…………	…………	May be a line of C_3_A·6H_2_O.
$[\begin{array}{l} 2.725\\ 2.654 \end{array}$^d^	14 4	c202 c211	$\begin{array}{l} 2.672\\ 2.669 \end{array}]$	…………	
2.524	18	$\{\;\begin{array}{c} 003\\ 300 \end{array}$	2.522 2.516	} 2.530	
2.488	18	…………	…………	…………	
$[\begin{array}{l} 2.441\\ 2.419 \end{array}$^d^	18 24	c103 c301	2.392 2.388	$\begin{array}{l} 2.430\\ 2.366 \end{array}]$	
2.339	22	…………	…………	…………	
2.295 2.238	4 4	} c212	2.276	…………	
2.165	6	$\{\;\begin{array}{c} 113\\ 220 \end{array}$	2.183 2.180	} 2.177	
2.116	8	…………	…………	2.118	Designated as 310 or 302 by Turriziani and Schippa.
2.096	6	$\{\;\begin{array}{c} 302\\ 203\\ 310 \end{array}$	2.097 2.093 …………	………… ………… …………	
2.065	2	…………	…………	…………	
2.024 2.012	2 6	} 311	2.018	2.023	
1.992	2	…………	…………	…………	
1.943	10	…………	…………	1.955	Designated as 004 by Turriziani and Schippa.
1.907	2	…………	…………	…………	
1.890	4	$\{\;\begin{array}{c} 004\\ 213\\ 222\\ 400 \end{array}$	1.891 1.889 1.888 1.887	………… ………… ………… …………	
1.861	8	…………	…………	…………	
1.824	12	$\{\;\begin{array}{c} {\;}^{c}104\\ {\;}^{c}312\\ {\;}^{c}401 \end{array}$	1.835 1.833 1.831	………… ………… …………	
$[\begin{array}{c} 1.661\\ 1.652\\ 1.642 \end{array}$^d^	12 16 12 …………	402 321 410 223	………… 1.688 1.648 …………	$\begin{array}{c} \ldots \ldots \ldots \ldots \\ 1.685\\ 1.665\\ \ldots \ldots \ldots \ldots \end{array}]$	
1.602	4	$\{\;\begin{array}{c} {\;}^{c}313\\ {\;}^{c}411 \end{array}$	1.611 1.610	………… …………	
1.580	4	$\{\;\begin{array}{c} 322\\ 214 \end{array}$	1.575 1.576	} 1.586	
1.512	6	$\{\;\begin{array}{c} 005\\ 304\\ 403\\ 412\\ 500 \end{array}$	1.513 1.512 1.511 1.510 …………	………… ………… ………… ………… …………	
1.436	4	$\{\;\begin{array}{c} 115\\ 224\\ 323\\ 420 \end{array}$	1.430 1.428 1.424 …………	………… ………… ………… …………	
1.410	2	$\{\;\begin{array}{c} {\;}^{c}205\\ {\;}^{c}314 \end{array}$	1.404 1.403	………… …………	
1.381	4	413	1.379	…………	

a *hkl* are assigned on the assumption of a hexagonal lattice. The expected *d*-spacings are calculated from the unit cell parameters *a*=8.716 and *c*=7.565, which were in turn calculated from the observed *d*-spacings. See discussions,

b See ref [3].

c See discussion in text concerning the *hkl* values marked.

d The bracketed *d*-spacings may correspond to one or several of the corresponding *hkl* values tabulated, and vice versa.

**Table 3:** Preparation of *3CaO·Al_2_O3·3CaCO_3_·32H_2_O*

Preparation No.	3.1	3.2	3.3

Total volume of mixture ml Original concentration of mixture, calculated:	1,000	1,000	1,000
CaO g/liter	1.94	3.41	6.16
Al_2_O_3_ g/liter	.46	.69	1.15
NH_4_HCO_3_ g/liter	.80	1.33	2.40
Sucrose g/liter	16	35	50
Final concentration of solution:^a^			
CaO g/liter	1.05	1.85	3.48
Molar ratios in precipitate (dried at 79% R.H.):			
CaO: Al_2_O_3_	5.90	5.88	6.00
CO_2_: Al_2_O_3_	……….	……….	3.02
H_2_O: Al_2_O_3_	……….	……….	31.9

a Other constituents not determined.

**Table 4:** X-ray powder diffraction patterns of *3CaO·Al_2_O_3_·3CaCO_3_·32H_2_O* and “silicoalaminate”

3CaO·Al_2_O_3_·3CaCO_3_·32H_2_O		“Calcium silicoaluminate”	

d	I	d	I

A		A	
9.41	100	9.69	100
5.43	27	5.56	25
5.31	4		
4.83	20	4.94	10
4.62	26	4.68	8
3.80	42	3.85	13
3.52	20	3.57	5
3.37	27	3.44	14
3.13	4	3.20	6
2.962	8	3.00	4
2.700	35	2.88	9
2.653	11	2.77	7
2.605	5	2.74	9
2.529	7	2.66	5
2.507	67	2.58	9
2.466	3	2.54	15
2.413	4		
2.339	6		
2.310	4	2.184	11
2.152	31	2.157	4
2.098	19	2.130	7
2.049	3	2.056	3
1.996	5	2.034	4
1.977	3		
1.913	7	1.948	3
1.879	12	1.917	5
1.822	9		
1.768	7	1.786	2
1.758	2		
1.739	2		
1.711	2	1.723	2
1.684	4		
1.646	4	1.665	3
1.622	7	1.645	2
1.582	2	1.597	2
1.568	2		
1.541	2	1.559	2
1.532	4		
1.483	5		
1.465	3		

## Footnotes

1 Figures in brackets indicate the literature reference at the end of this paper.
